# Focusing on Future Consequences Enhances Self-Controlled Dietary Choices

**DOI:** 10.3390/nu16010089

**Published:** 2023-12-27

**Authors:** Johanna Kruse, Franziska M. Korb, Caroline Surrey, Uta Wolfensteller, Thomas Goschke, Stefan Scherbaum

**Affiliations:** Department of Psychology, TUD Dresden University of Technology, 01069 Dresden, Germany; franziska.korb@tu-dresden.de (F.M.K.); caroline.surrey@tu-dresden.de (C.S.); uta.wolfensteller@tu-dresden.de (U.W.); thomas.goschke@tu-dresden.de (T.G.); stefan.scherbaum@tu-dresden.de (S.S.)

**Keywords:** food choice, decision making, self-control, mouse tracking, process tracing

## Abstract

Self-controlled dietary decisions, i.e., choosing a healthier food over a tastier one, are a major challenge for many people. Despite the potential profound consequences of frequent poor choices, maintaining a healthy diet proves challenging. This raises the question of how to facilitate self-controlled food decisions to promote healthier choices. The present study compared the influence of implicit and explicit information on food choices and their underlying decision processes. Participants watched two video clips as an implicit manipulation to induce different mindsets. Instructions to focus on either the short-term or long-term consequences of choices served as an explicit manipulation. Participants performed a binary food choice task, including foods with different health and taste values. The choice was made using a computer mouse, whose trajectories we used to calculate the influence of the food properties. Instruction to focus on long-term consequences compared to short-term consequences increased the number of healthy choices, reduced response times for healthy decisions, and increased the influence of health aspects during the decision-making process. The effect of video manipulation showed greater variability. While focusing on long-term consequences facilitated healthy food choices and reduced the underlying decision conflict, the current mindset appeared to have a minor influence.

## 1. Introduction

Many people are challenged with food-related self-controlled decision-making. Self-control is the ability to choose a larger delayed reward over a smaller immediate one [1,2,3]. Transferred to food-related decisions, this implies that self-control means choosing the healthier option, whose benefits will occur later in time, instead of the tastier option with its comparable smaller but immediate reward. One possible outcome of frequent poor dietary decision-making is obesity, which goes along with physical, psychosocial, and economic consequences such as hypertension, type 2 diabetes mellitus, depression, anxiety, and profuse health care expenditures (for an overview, see [4,5]). However, frequent and unsuccessful efforts to stay on a diet show that people seem to be aware that healthier food intake is the better choice. Yet they seem to have trouble making decisions in accordance with this goal [6]. So, why are healthy food choices difficult for many people, and how can dietary self-controlled behavior, namely rejecting the short-term tastier option and choosing the long-term healthier option instead, be improved? These questions are not only relevant on an individual level but also provide the foundation for government policies on how to encourage people to maintain healthier lifestyles [7].

### 1.1. Influences on Dietary Decisions

Food choices are complex decisions that can be influenced by various aspects. Two important determinants are the actual and/or perceived healthiness and tastiness of food items, which have been of interest in prior research [8,9,10]. From a neuroscience perspective, the brain makes decisions by computing and comparing the values of different stimulus properties, e.g., while choosing a food item that has different taste and health values [11,12,13]. While food with a comparatively high taste value is associated with an immediate reward and thus represents a temptation, food with a higher health value would correspond to the long-term goal of a healthy diet. Due to this difference, those decisions imply a self-control conflict. Whereas people with higher self-control take both types of information into account, people with lower self-control give more weight to the taste information and therefore choose the healthy option less often [14]. Moreover, basic attributes like taste are processed faster than more abstract attributes, like healthiness, and therefore have an earlier impact on the choice process [15]. However, previous research has shown that various factors, such as attentional aspects [16,17], monetary incentives [10], external health-related cues [18], and a person’s current mindset [19,20,21], can influence food choices towards healthier choices.

### 1.2. Mouse-Tracking as a Process-Tracing Method

**Figure 1:**
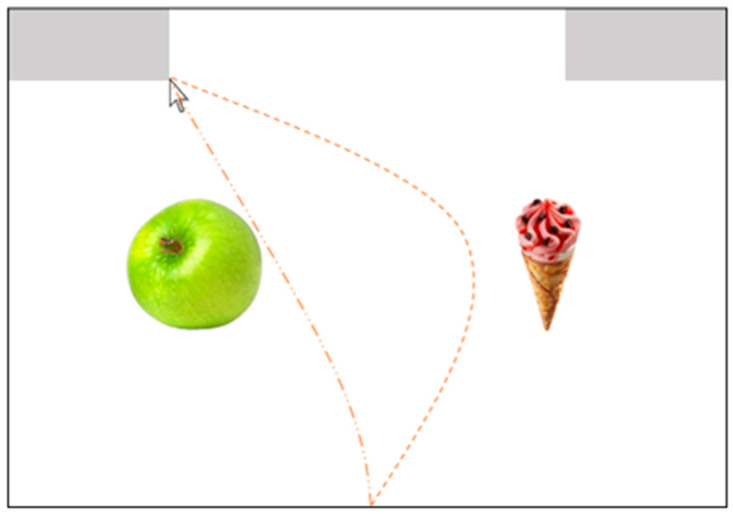
Examples of mouse movements during one trial. The dotted line shows an example of a mouse movement, where one first tends to choose the ice cream and only later decides in favor of the apple. The dash-dotted line is an example of a mouse movement in which the apple is selected directly.

When investigating influences on food choices, it is important to consider the process through which a certain factor affects food choices. One method to uncover underlying decision processes is mouse-tracking, the movement tracking of a person’s computer mouse during a decision [22,23]. Mouse-tracking has proved to be a valuable method to examine dietary self-control conflicts [8,15,18,24,25]. During a mouse-tracking task, participants are instructed to indicate their response using a computer mouse. They start moving from a starting field in the bottom center to pre-defined choice fields in the upper corners of the screen. The underlying assumption is that the choice process has an impact on hand movements and hence is traceable within a trial during the movement of the computer mouse from start to respective choice-field [23]. For instance, when faced with a choice between an apple and ice cream, one may initially lean towards the latter option and move the cursor in that direction (see Figure 1). However, upon considering dietary restrictions or health concerns, the decision-maker may reconsider their initial choice and ultimately opt for the apple, leading to a deviation from the previously favored option. Hence, greater deflection is associated with more conflict during the decision-making process. This information allows for more insight into the underlying decision-making process compared to static measures like choice times.

### 1.3. Aims of the Current Study

Previous research has shown that both specific information or incentives as well as differing mindsets have an impact on dietary self-control [10,18,21]. However, it remains unclear how the mechanisms of the various influences on dietary decisions and their underlying processes differ. Does a general mental framework sufficiently support acting according to long-term goals? Or does it need an explicit, constant reminder at the moment of decision? Moreover, how do different types of information affect the underlying decision conflict? This knowledge could be useful in designing effective interventions for individuals or even in developing strategies to encourage a population to adopt healthy lifestyles.

Hence, in the current study, we compare two potential influences on food choices. Firstly, as an explicit manipulation, we compared the influence of attentional focus on either short-term or long-term consequences. Secondly, as a more implicit manipulation, we compared the influence of a mindset towards a balanced diet and a mindset with unrelated content. We expected that the mindset towards a balanced diet (implicit manipulation) and the focus on the long-term consequences of food choices (explicit manipulation) would promote choices of healthy food items, whereas the explicit manipulation should have stronger effects compared to the implicit manipulation. While focusing on long-term consequences, we expected the positive effects of healthy food choices to be enhanced. Therefore, healthiness is considered more than tastiness in the decision-making process, which would make it easier to implement long-term goals and reject the tastier food option in favor of the healthier option. Furthermore, the enhanced influence of health aspects should also be reflected in mouse movements.

**Experiment 1**

## 2. Materials and Methods—Experiment 1

### 2.1. Data Statement and Availability of Data and Materials

We report how we determined our sample size, all data exclusions (if any), all manipulations, and all measures in this study. The datasets and analysis scripts supporting the conclusions of this article are openly available in the OSF repository, https://osf.io/3n42s/ (accessed on 26 December 2023).

### 2.2. Participants

Participants were recruited using the ORSEE-based database [26] of the Faculty of Psychology of the Dresden University of Technology, Dresden, Germany. The inclusion criteria were as follows: 18–35 years of age, no allergies or food intolerance, no vegetarian or vegan diet, no weight-loss-oriented restricting diet, no dyschromatopsia, and no cannabis consumption the day before the experiment. The sample size was based on previous work using within-subjects designs and a mouse-tracking task [15]. In total, 97 participants were recruited (62 female, mean age = 24.56 years, *SD* = 8.23 years). Due to the item combinations in the decision task, the ratings had to follow a certain distribution. For example, a certain minimum number of healthy, less tasty items was required (for details, see Section 2.5.1). For 45 participants, all cells for the necessary item combinations were filled so that these people could continue with the decision task. Of those, two participants were excluded due to a vegetarian diet, one participant was excluded because of technical difficulties, and one participant did not meet our age criteria. The final sample that entered the main experiment consisted of 41 participants (30 female, mean age = 22.22 years, *SD* = 6.15 years). Participants were either paid 8 €/h or got course credit for participation. They were informed that they might only be able to participate in the first part of the experiment. In this case, the time required was rounded up to 30 min, and the participants were paid accordingly. All participants had normal or corrected-to-normal vision. Prior to the experiment, they gave informed consent. This study was performed in accordance with the Declaration of Helsinki and was approved by the ethics committee of Dresden University of Technology, Germany (IRB00001473/EK47022016).

### 2.3. Apparatus and Stimuli

The experiment was presented on a 17-inch screen (1280 × 1024 pixels, 75 Hz). As presentation software, the Psychophysics Toolbox 3 [27] in Matlab 2010b (the Mathworks Inc., Natick, MA, USA) was used, running on a Windows XP SP2 personal computer. Responses were carried out by moving a high-precision computer mouse (Logitech Laser Mouse USB, Logitech, Suzhou, China). As stimuli, a selection of food images (300 × 225 pixels) from the Food-pics image database was used [28]. Self-created line drawings of a paper airplane and a rocket (both 256 × 192 pixels) served as cues for the current consequences to be considered during the decision task (for details, see Section 2.5.2). Both cues were comparable in simplicity and number of lines. Furthermore, two short video clips were presented in the decision task. One was about components of food and the importance of a balanced diet (3:26 min; in the following referred to as ‘nutrition’-video clip; https://www.youtube.com/watch?v=yM7NrKNy6K0 (accessed on 26 December 2023)) and therefore indirectly related to food choices, the other one about myths and reasons for headaches and migraines (4:10 min; in the following referred to as‘migraine’—video clip; https://www.youtube.com/watch?v=dtYxlEHXpdA (accessed on 26 December 2023)), and thus, with food-choice unrelated content. Both videos were freely available on YouTube and comparable in style and length, as the content was explained by the same person in the same setting.

### 2.4. Procedure

#### 2.4.1. Study Procedure

First, participants gave informed consent and answered a short pre-experiment questionnaire about their age, sex, and current state of health and mood. They also saw a printed overview of all the food items used in the experiment.

The experiment consisted of two parts. In the first part, participants were asked to rate 192 food items according to their perceived tastiness and healthiness by sorting them into boxes based on a five-point Likert-Scale from −2 (barely tasty/healthy) to 2 (very tasty/healthy). The rating of tastiness and healthiness was performed consecutively in two blocks, whereas the order was randomized across the participants. During the rating, participants saw a picture, the name of the respective item, and the rating boxes on the screen. In each rating box, the name of the last item that had been sorted into that box was visible. A box was empty if no item had been assigned to it. The variety of food items ranged from vegetables, fruits, chocolate, and candy to processed foods like hamburgers and pizza, covering all dietary options.

In the second part of the task, participants made decisions as to which of two food items they would prefer to eat and indicated their choice by using a computer mouse. The decision task consisted of four blocks of 60 trials each (for details, see Section 2.5.1). Before the first and third blocks, participants saw a short video clip (for details, see Section 2.3).

After the decision task, participants answered a short post-experimental questionnaire about their current state and mood and about their beliefs regarding the presumed aim of this experiment. In total, the experiment lasted approximately 40 min.

#### 2.4.2. Decision Task Procedure

**Figure 2:**
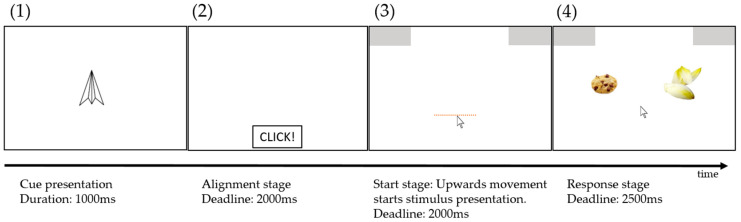
Stages of trial presentation during the decision task. After cue presentation (1), participants had to click in the start box at the bottom center of the screen (2) and move upwards at least 50 pixels (dashed line) (3) to start stimulus presentation (4). Participants had up to 2.5 s to decide on one of the two food items.

In each trial, an individualized pair of food items was presented based on the rating in the first part (for details, see Section 2.5.1). Each trial consisted of four stages (see Figure 2). First, the relevant cue for the subsequent trial was shown in the center of the screen for one second. Then, a box appeared at the bottom center of the screen. Participants had to click in this box within a time limit of two seconds in order to ensure a comparable starting area for each trial. After clicking in this box, two response boxes appeared in the upper right and upper left corners of the screen, and participants had to start the mouse movement within two seconds. Using this procedure, participants were forced to already be moving when entering the decision process, and we assured them that they did not decide first and then just performed the final movement [29,30]. After moving at least 50 pixels, the target stimulus appeared in the center of the screen. Participants chose one of the two items by moving the cursor to the corresponding response box within a time limit of 2.5 s. In case participants missed one of the three deadlines (i.e., alignment, start stage, or response stage), the next trial started after 0.5 s with the presentation of the cue. Trials with a missed deadline remained in the trial pool and were presented again in a random order. Response times represent the time between the onset of the target stimulus and reaching the response box with the mouse cursor. Participants were familiarized with the mouse tracking paradigm during a tutorial. After on-screen instructions, the correct movement was explained and demonstrated by the experimenter. Participants then performed 16 trials with feedback from the experimenter and no time limit, and 16 trials without feedback and with a time limit.

### 2.5. Design

#### 2.5.1. Task Design

**Figure 3:**
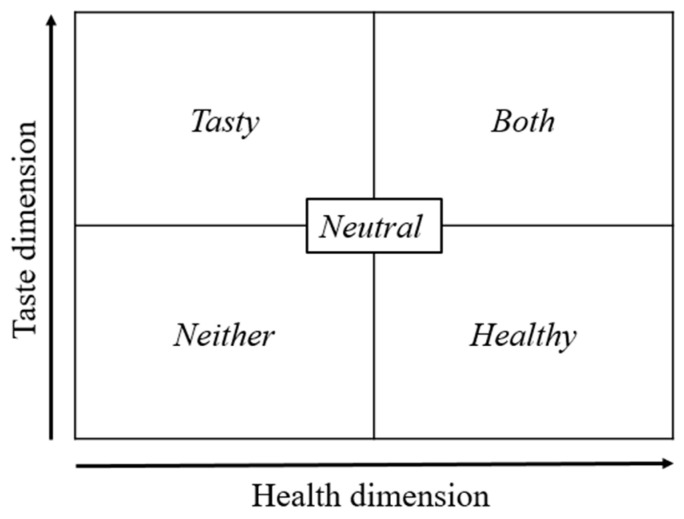
Food item categories. Each item was post-hoc categorized as either “Tasty” (less healthy and tasty), “Both” (healthy and tasty), “Neither” (less healthy and less tasty), “Healthy” (healthy and less tasty), or “Neutral” (neutral in both healthy and tasty). One neutral item was chosen as a reference item.

Based on the individual ratings in the first part, food items were categorized for each participant as either healthy and less tasty (*healthy*), less healthy and tasty (*tasty*), healthy and tasty (*both*), less healthy and less tasty (*neither*), or neutral in both healthy and tasty (*neutral*) (see Figure 3). One *neutral* item was randomly chosen as a reference item. Due to trial combinations in the decision task (see below), each participant had to have at least ten *healthy* food items, ten *tasty* food items, five items that were *both* healthy and tasty, five items that were *neither* healthy nor tasty, and one *neutral* food item to take part in the decision task.

The decision task included 40 conflict trials requiring self-control and 20 control trials with an objectively correct answer. Trials were randomly presented in four blocks, whereas the order of the trials differed in each block. In total, the decision task comprised 240 trials. Conflict trials were created in the following way: ten trials were created by combining ten *healthy* items with the reference item, ten trials by combining ten *tasty* items with the reference item, and twenty trials by combining *healthy* items with *tasty* items in twenty different combinations. Control trials were created by combining five items, which were *both* healthy and tasty (see Figure 3); with five *healthy* items; five items, which were *both* with five *tasty* items; furthermore, five items, which were *neither* healthy nor tasty, with five *healthy* items; and last but not least, five items, which were *neither* healthy nor tasty, with five *tasty* items.

#### 2.5.2. Study Design

We manipulated self-control using a block-wise 2 (implicit: video manipulation) ×2 (explicit: instruction manipulation) factorial within-participants design. The video manipulation served to implicitly manipulate self-controlled behavior. Participants saw a short video clip before the first and third blocks of the experiment, either about components of food and the importance of a balanced diet (self-control condition) or about myths and reasons for headaches and migraines (neutral condition). Thereby, we induced different mindsets for each half of the experiment. The order of the video clip presentation was randomized across participants. The instruction manipulation served to explicitly manipulate self-controlled behavior. Participants saw on-screen instructions to focus on either short- or long-term consequences of their decision at the beginning of each block, two of which were presented within each experiment half. Additionally, a cue was shown before each trial in the respective block as a reminder. Short-term consequences were illustrated by an image of a paper airplane (which can only fly short distances), whereas long-term consequences were illustrated by a rocket (which can travel long distances). The meaning of the cues was explained to the participants in the tutorial. Block instructions alternated within the experiment, whereas the type of the first block per experiment half was randomized across participants but stayed constant within each half, i.e., per video clip condition.

#### 2.5.3. Data Processing

Data pre-processing, processing, and analysis of the mouse trajectories were performed using Matlab 2018b together with the TCMR Toolbox [31] running on a Windows 10 computer. Statistical analysis was performed using JASP version 0.10.2.0 [32].

### 2.6. Data Pre-Processing

#### Consistency Check

To exclude participants who made random choices, we examined choices in control trials where participants had to choose either between an item that was *both* healthy and tasty and a *healthy* item (with the item that was *both* the correct choice) or between a *tasty* item and an item that was *neither* healthy nor tasty (with the *tasty* item as the correct choice). On average, participants made the correct choice in 84.2% (*SD* = 12.18%). Two participants were identified as outliers due to their low number of correct choices (47.5% and 55%, respectively) and were subsequently excluded from further analyses.

### 2.7. Mouse Trajectories

Pre-processing of mouse trajectories was performed based on [31]. Mouse trajectories were aligned for mutual starting positions (horizontal center of the screen, 640 pixels). Movement dynamics were analyzed based on the trajectory angle on the XY plane. We calculated the trajectory angle as the angle relative to the Y-axis for each difference vector delta-X and delta-Y between two time steps.

Before analyzing mouse trajectories, we checked the consistency of mouse movements by examining continuity (i.e., how straight the upward movement was) and the amount of returns (i.e., how many backward movements occurred) for all participants [31]. Four participants were identified as outliers due to their high number of returns during conflict trials (more than 50%). Therefore, those participants were excluded from the analysis of mouse movement data, leaving a sample size of 35 participants.

The following procedure was applied for calculating the time-continuous multiple regression (TCMR): at first, two predictors were coded for each trial for all participants. One predictor coded the difference in health-values between the two options to capture the influence of the health dimension on the mouse movement. The other one coded the difference in taste-values between the two options to capture the influence of the taste dimension on the mouse movement. Afterwards, both predictors were normalized to a range from −1 to 1 to provide comparable beta-weights. In the last step, multiple regressions were calculated with the normalized predictors on the data from each time slice of the trajectory angle for all four condition-combinations (i.e., instruction × video). The trajectory angle was also normalized to −1 and 1 for each participant. This resulted in two time-varying beta-weights for all conditions for each participant.

### 2.8. Data Analysis

#### 2.8.1. Behavioral Data

To test for statistical differences in self-controlled choice behavior between conditions, a repeated measure analysis of variance (rmANOVA) with the factors *Video* (nutrition; migraine) and *Instruction* (short-term consequences; long-term consequences) was performed. Response times were tested for significant differences between conditions using a rmANOVA with the following factors: *Video* (nutrition; migraine), *Instruction* (short-term consequences; long-term consequences), and *Choice* (healthy; unhealthy). Even though the order of the video was counter-balanced across participants, we checked for possible transfer effects due to the order of the videos. To this end, we calculated an additional rmANOVA with the within factors *Instruction* (short-term consequences; long-term consequences) and *Video* (nutrition; migraine) and the between factor *Video order* (1st nutrition, 2nd migraine; 1st migraine, 2nd nutrition).

#### 2.8.2. Time-Continuous Multiple Regression

To detect significant temporal segments of influence, we calculated the contrast of beta-weights by subtracting the beta-weights of the health difference predictor from the beta-weights of the taste difference predictor. Then, we computed one-sample *t*-tests for each time step of this contrast-measure. Ref. [33] showed that correction for multiple comparisons can be realized by only accepting segments of more than ten sequential *t*-tests.

## 3. Results—Experiment 1

### 3.1. Choice Behavior

**Figure 4:**
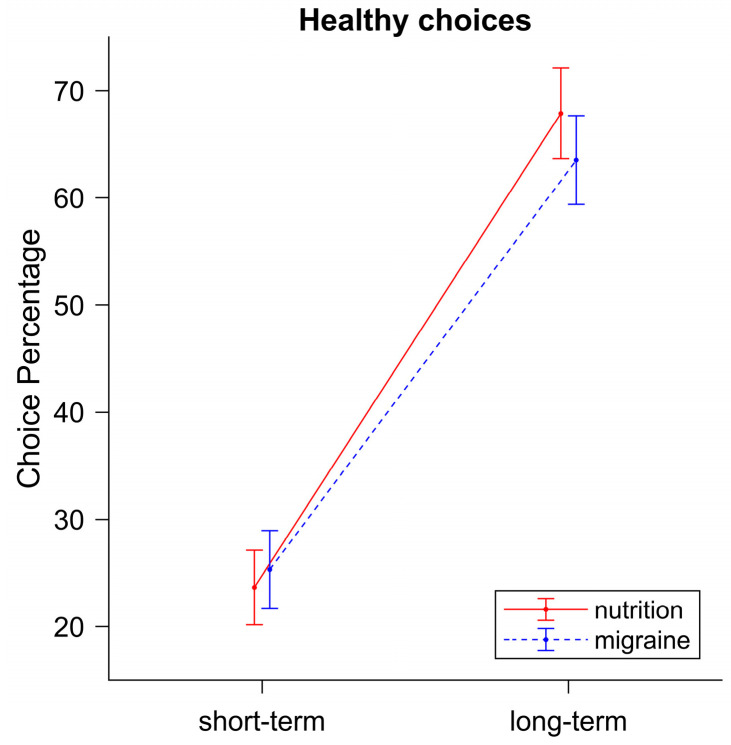
Percentage of healthy choices following short-term vs. long-term instructions after watching food-related (nutrition) or unrelated (migraine) videos. When focusing on long-term consequences, significantly more healthy choices were made in the nutrition video-block compared to the migraine video-block. Error bars display standard errors.

As expected, we found a significant main effect for *Instruction*: *F*(1,38) = 119.35, *p* < 0.001, η^2^ = 0.43. Participants chose the healthy option significantly more often when they were instructed to focus on the long-term consequences of their choices (65.71%) as compared to the short-term consequences (24.49%), which is in accordance with our hypothesis. The main effect for *Video* was not significant, *F*(1,38) = 0.96, *p* = 0.33, η^2^ = 0.00, but we found a significant interaction effect for *Instruction ∗ Video*, *F*(1,38) = 5.82, *p* = 0.02, η^2^ = 0.002. Partly in line with our hypothesis, participants made significantly more healthy choices during the long-term condition when they watched a short video-clip about nutrition (67.88%) as compared to a short video-clip about migraine (63.52%, see Figure 4).

The analysis of possible transfer effects due to the order of videos showed that the order had no influence on any of the significant results reported above and can thus be neglected.

### 3.2. Response Times

**Figure 5:**
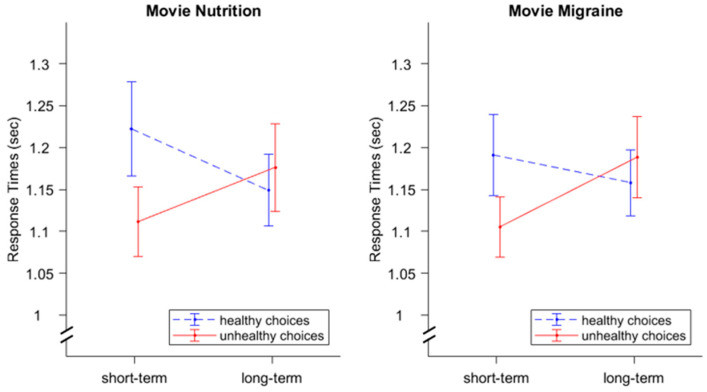
Response times for both videos (nutrition and migraine) while focusing on the short-term vs. long-term consequences. For short-term instructions, smaller response times were found for unhealthy choices (as compared to healthy choices). For long-term instructions, smaller response times were found for healthy choices (as compared to unhealthy choices). The difference between healthy and unhealthy choices was bigger for short-term instructions compared to long-term instructions. This pattern was found for both videos. Error bars display standard errors.

We found a significant interaction effect for *Instruction ∗ Choice*, *F*(1,33) = 15.72, *p* < 0.001, η^2^ = 0.01 (see Figure 5). Response times were smaller for healthy choices when participants focused on the long-term consequences (1.15 s) as compared to short-term consequences (1.21 s), in contrast to response times during unhealthy choices, which were higher under the condition of long-term-consequences (1.18 s) as compared to short-term consequences (1.11 s). We did not find an effect for *Video*; *F*(1,33) = 0.67, *p* = 0.42, η^2^ = 0.00. The current mindset does not appear to affect choice times. Furthermore, no significant effects were found for *Instruction*, *F*(1,33) = 0.68, *p* = 0.42, η^2^ = 0.00, *Choice*, *F*(1,33) = 3.01, *p* = 0.09, η^2^ = 0.00, *Video ∗ Instruction*, *F*(1,33) = 0.27, *p* = 0.61, η^2^ = 0.00, *Video ∗ Choice*, *F*(1,33) = 0.00, *p* = 0.99, η^2^ = 0.00, and *Video ∗ Instruction ∗ Choice*, *F*(1,33) = 32, *p* = 0.58, η^2^ = 0.00.

### 3.3. Time-Continuous Multiple Regression on Mouse Movement Angle

**Figure 6:**
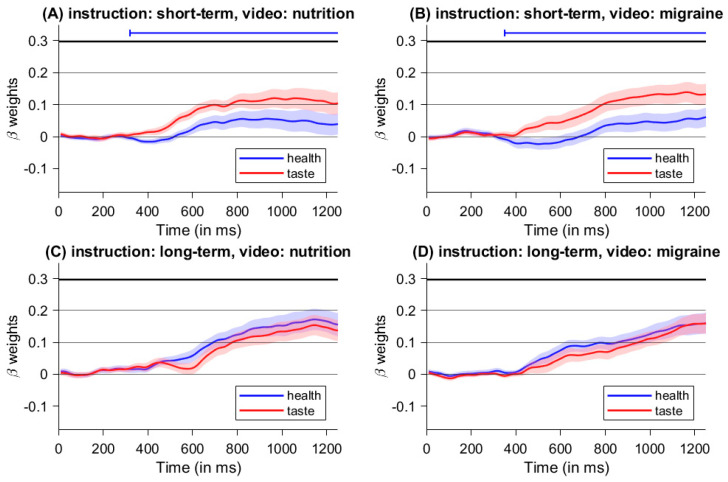
Stimulus-locked time-continuous beta-weights from time-continuous multiple regression of Experiment 1 for all four manipulation combinations. The lines above the graphs mark significant segments determined by *t*-tests against zero. Significant differences between both predictors were found for short-term instructions in both videos.

For short-term instructions (emphasizing taste) following a nutrition video (healthy mindset), we found significant differences between the beta-weights of taste-differences and health-differences for trials with short-term instructions and watching the nutrition-video clip between 320 ms and 1200 ms after stimulus onset (see Figure 6A). Beginning at 320 ms, mouse angle trajectories were significantly more influenced by taste properties compared to the health properties of the food items. Similarly, for short-term instructions (emphasizing taste), there were significant differences between the beta-weights of taste-differences and health-differences for trials with short-term instructions and watching the migraine-video clip between 340 ms and 1200 ms after stimulus onset (see Figure 6B). From 340 ms on, mouse angle trajectories were also significantly more influenced by taste properties compared to the health properties of the food items. No significant differences were found during long-term instructions for both videos (see Figure 6C,D). Table 1 shows all time segments with consecutive beta weights.

As expected, those results show that taste properties have a greater influence on the decision process than health properties during the short-term condition. Furthermore, the absence of a significant difference during long-term instructions for both videos suggests that the influence of health properties was enhanced during long-term instructions.

The results of Experiment 1 might be influenced by a potential priming effect of instructions due to block-wise presentations of the respective cue (thereby possibly creating a second mindset intervention that overrides the effects of our actually intended mindset manipulation by the video clip). In order to exclude this potential influence, we conducted a second experiment with randomized instruction cues for each trial instead of the block-wise instruction manipulation.

**Experiment 2**

## 4. Material and Methods—Experiment 2

Apart from the differences described in the following, Experiment 1 and Experiment 2 were identical.

### 4.1. Participants

In total, 73 participants were recruited (47 female, 24 male, 2 with no information, mean age = 22.74 years, *SD* = 4.33 years); for 42 participants, all cells for the necessary item combinations were filled so that these persons could continue with the decision task (see Section 2.5.1); two participants were excluded due to technical difficulties, and one participant did not have sufficient German language skills. The final sample that completed the main experiment consisted of 39 participants (26 female, 11 male, 2 with no information, mean age = 22.51 years, *SD* = 4.36 years).

### 4.2. Apparatus and Stimuli

In this experiment, colored frames (orange and purple) around the food stimuli served as cues during the decision task.

### 4.3. Procedure

#### Decision Task Procedure

**Figure 7:**
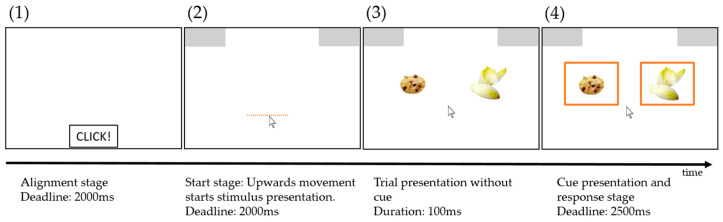
Stages of trial presentation during the decision task. Participants had to click in the start box at the bottom center of the screen (1) and move upwards at least 50 pixels (dashed line) (2) to start the stimulus presentation (3). The cues appeared after 100 ms (4). Participants had up to 2.5 s to decide on one of the two food items.

The decision-task procedure differed from Experiment 1 only in terms of cue presentation. Each trial consisted of four stages (see Figure 7). First, a box appeared at the bottom center of the screen. Participants had to click in this box within a time limit of two seconds in order to ensure a comparable starting area for each trial.

After clicking in this box, two response boxes appeared in the upper right and upper left corners of the screen, and participants had to start the mouse movement within a deadline of two seconds. After moving at least 50 pixels, the two target stimuli appeared left and right from the center of the screen on the vertical midline, followed by the instruction cue for the respective trial after 100 ms. As in Experiment 1, participants chose one of the two items by moving the cursor to the corresponding response box within 2.5 s.

### 4.4. Design

#### Study Design

We manipulated self-control using a 2 (implicit: video manipulation) × 2 (explicit: instruction manipulation) factorial within-participants design. Similar to Experiment 1, a short video clip (either about nutrition or migraine) was shown before each half of the experiment. In Experiment 2, a randomized cue (orange vs. purple frame) was shown during each trial. Participants were instructed to focus on the short-term consequences of their choices in the respective trial when an orange frame appeared around the food items. When a purple frame appeared, participants were instructed to focus on the long-term consequences of their decision.

### 4.5. Data Pre-Processing

#### 4.5.1. Consistency Check

Similar to Experiment 1, we ruled out that participants made random choices by examining choices in control trials where participants had to choose either between an item that was *both* and a *healthy* item (with the item that was *both* the correct choice) or a *tasty* item and an item that was *neither* (with the *tasty* item as the correct choice). On average, participants made the correct choice in 81.09% (*SD* = 14.28%). There were no outliers in Experiment 2.

#### 4.5.2. Mouse Trajectories

Equivalent to Experiment 1, we checked the consistency of mouse movements by examining continuity (i.e., straightness of upward movement) and the amount of returns (i.e., backward movements) for all participants [31]. Two participants were identified as outliers due to their high number of returns during conflict trials (in excess of 50%) and therefore excluded from the analysis of mouse movement data. Two further participants were excluded due to too high Variance Inflation Factors, which indicates multicollinearity of regressors in multiple regression analysis. This left a sample size of 35 participants for the analysis of mouse movement data.

## 5. Results—Experiment 2

### 5.1. Choice Behavior

**Figure 8:**
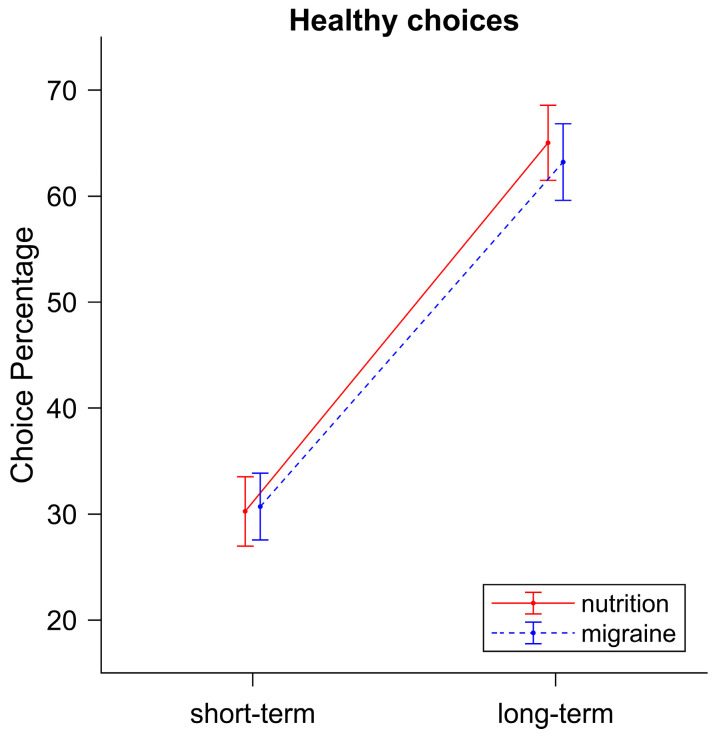
Percentage of healthy choices following short-term vs. long-term instructions after watching food-related (nutrition) or unrelated (migraine) videos.

The rmANOVA revealed a significant main effect for *Instruction*: *F*(1,38) = 35.15, *p* < 0.001, η^2^ = 0.30. Participants made significantly more healthy choices when they were instructed to focus on the long-term consequences of their choices (64.12%) as compared to the short-term consequences (30.48%, see Figure 8), which is in accordance with our hypothesis. In contrast to Experiment 1, the *Instruction ∗ Video* interaction effect were not significant (*F*(1,38) = 0.42, *p* = 0.52, η^2^ = 0.00), but descriptively, the data displayed the same pattern. The main effect for *Video* was also not significant: *F*(1,38) = 0.13, *p* = 0.72, η^2^ = 0.00.

Similar to the first experiment, the order of the videos had no influence on the above-mentioned significant results.

### 5.2. Response Times

**Figure 9:**
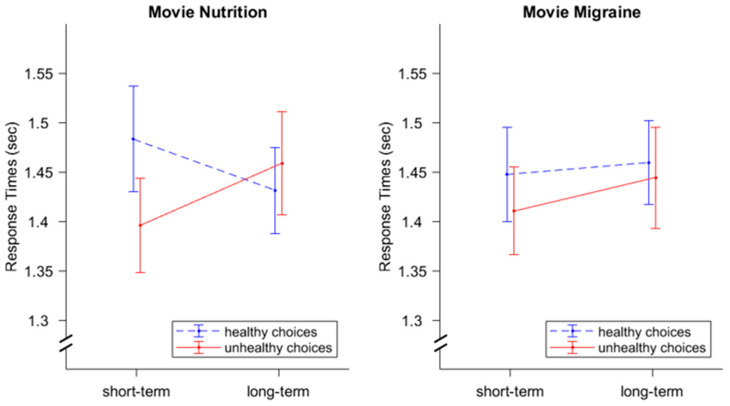
Response times for both videos (nutrition and migraine) while focusing on the short-term vs. long-term consequences. With regard to the nutrition video, smaller response times were found for short-term instructions and unhealthy choices (as compared to healthy choices). Long-term instructions were found to have shorter reaction times for healthy decisions (compared to unhealthy decisions). The difference between healthy and unhealthy choices was bigger for short-term instructions compared to long-term instructions. No significant effects were found for the migraine video. Error bars display standard errors.

Similar to Experiment 1, we found a significant interaction effect for *Instruction ∗ Choice*, *F*(1,37) = 5.73, *p* = 0.022, η^2^ = 0.004. Furthermore, we found a significant interaction effect for *Instruction ∗ Video ∗ Choice*, *F*(1,37) = 5.36, *p* = 0.026, η^2^ = 0.002 (see Figure 9). No other effects were found to be significant (*Instruction*, *F*(1,37) = 1.70, *p* = 0.20, η^2^ = 0.00, *Video*: *F*(1,37) = 0.00, *p* = 0.98, η^2^ = 0.00, *Choice*: *F*(1,37) = 2.53, *p* = 0.12, η^2^ = 0.00, *Instruction ∗ Video*, *F*(1,37) = 0.97, *p* = 0.33, η^2^ = 0.00; *Video ∗ Choice*, *F*(1,37) = 0.02, *p* = 0.89, η^2^ = 0.00.

Follow-up 2 × 2 factorial rmANOVA with the within factors *Instruction* (short-term consequences; long-term consequences) and *Choice* (healthy; unhealthy) for both videos separately revealed a significant interaction effect for *Instruction ∗ Choice*, *F*(1,38) = 6.35, *p* = 0.016, η^2^ = 0.01 for the nutrition video but not for the migraine video, *F*(1,37) = 0.73, *p* = 0.40, η^2^ = 0.00. Main effects for *Instruction* and *Choice* were not significant in either analysis (nutrition video: *Instruction*, *F*(1,38) = 0.13, *p* = 0.72, η^2^ = 0.00, *Choice*, *F*(1,38) = 2.45, *p* = 0.13, η^2^ = 0.00; migraine video: *Instruction*, *F*(1,37) = 2.32, *p* = 0.14, η^2^ = 0.00, *Choice*, *F*(1,37) = 1.78, *p* = 0.19, η^2^ = 0.00).

A post-hoc analysis indicated that the three-way interaction effect was based on an *Instruction ∗ Choice* interaction only during the nutrition video. With a relevant mindset intervention (i.e., nutrition video), the cue led to faster unhealthy choices (1.40 s) compared to healthy choices (1.48 s) during the short-term instruction. In contrast, for the long-term instructions, choice times were smaller for healthy choices (1.43 s) compared to unhealthy choices (1.46 s).

Hence, those results partially confirmed our hypotheses that participants will make faster choices if their choice is congruent to the respective instruction, but only for the nutrition video condition. Contrary to our expectations, we did not find a main effect for *Video*.

### 5.3. Time-Continuous Multiple Regression on Mouse Movement Angle

**Figure 10:**
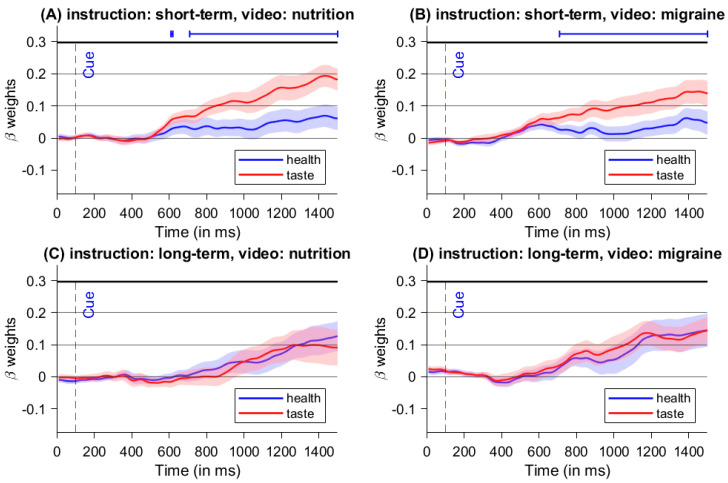
Stimulus-locked time-continuous beta-weights from time-continuous multiple regression of Experiment 2 for all four manipulation combinations The lines above the graphs mark significant segments determined by *t*-tests against zero. Significant differences between both predictors were found for short-term instructions in both videos.

We found significant differences between the beta-weights of taste-differences and health-differences for trials with short-term instructions and watching the nutrition-video clip between 610 ms and 620 ms and from 710 ms after stimulus onset on (see Figure 10A). Between those time segments, mouse angle trajectories were significantly more influenced by taste properties compared to the health properties of the food items. Furthermore, there are significant differences between the beta-weights of taste-differences and health-differences for trials with short-term instructions and watching the migraine-video clip from 710 ms after stimulus onset onwards (see Figure 10B). Starting at 710 ms, mouse angle trajectories were also significantly more influenced by taste properties compared to the health properties of the food items. No significant differences were found during long-term instructions for both videos (see Figure 10C,D). An overview of all time segments with consecutive significant beta weights can be found in Table 2.

Similar to Experiment 1, we found a higher influence of taste properties compared to health properties on the decision process during short-term instructions, with a comparable pattern for both video conditions. However, the duration of the significant difference was shorter than in the first experiment. These results support our hypothesis that food choices are especially influenced by taste while focusing on short-term consequences. Just as in the previous experiment, we did not find a significant difference during long-term instructions for both videos. Furthermore, results suggest that the influence of health properties can be enhanced while focusing on long-term consequences, which is also in line with our hypothesis. But the influence of health properties did not exceed the influence of taste properties on the decision process. Contrary to our expectations, we did not find differences between distinct video conditions.

**Figure 11:**
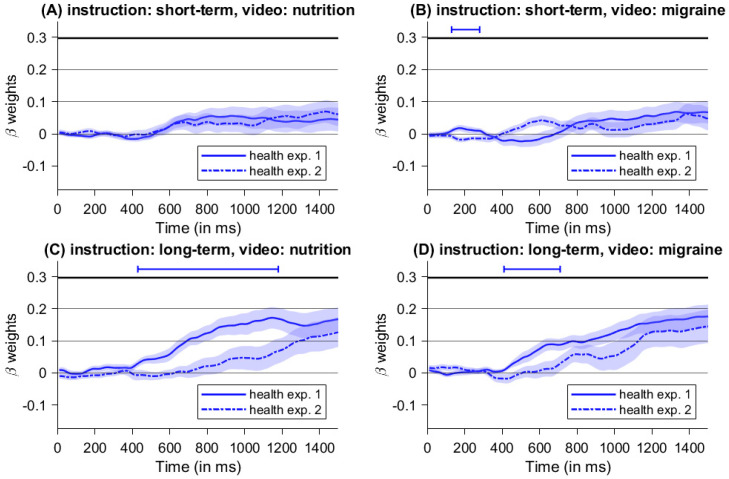
Comparison of beta weights of health differences between experiments, with blocked cue presentation in Experiment 1 and random cue presentation in Experiment 2. The lines above the graphs mark significant segments determined by *t*-tests against zero.

**Figure 12:**
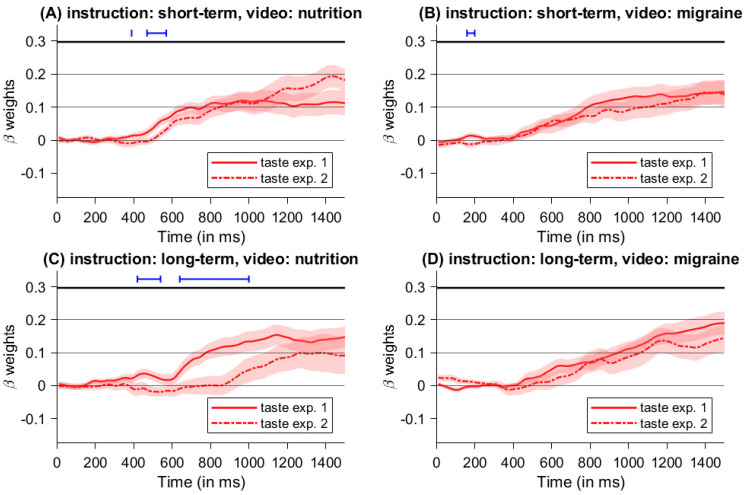
Comparison of beta weights of taste differences between experiments, with blocked cue presentation in Experiment 1 and random cue presentation in Experiment 2.

In summary, both experiments showed a high influence of instructed consequences on dietary decisions, while we found no significant difference between video manipulations. However, the type of cue presentation (i.e., blocked versus randomized) might have different influences on the decision process. Hence, we compared beta weights of health differences and taste differences between both experiments (see Figure 11 and Figure 12).

Regarding the influence of health properties (see Figure 11), we found no significant beta-weight differences between experiments for trials with short-term instructions and watching the nutrition-video clip (see Figure 11A). For decisions with short-term instructions following the migraine video, the influence of health properties was significantly different between 130 and 280 ms (see Figure 11B). The biggest difference between health properties was found for long-term instructions and the nutrition-video (between 430 ms and 1180 ms, see Figure 11C), whereas the window of significant differences for long-term instructions and the migraine-video was between 410 ms and 710 ms (see Figure 11D), indicating a stronger influence of cue presentation type during long-term instructions. For an overview, see Table 3. While there is little or no difference for decisions with short-term instructions, blocked cue presentation (as in Experiment 1) enhances the influence of health properties compared to random cue presentation (as in Experiment 2) for trials with instructed long-term consequences.

The influence of taste properties significantly differed between experiments for decisions with short-term instructions following the nutrition video at 390 ms and between 470 ms and 570 ms after stimulus onset (see Figure 12A) and for decisions with short-term instructions following the migraine video between 160 ms and 200 ms (see Figure 12B). Again, the largest time frame with a significant difference was on those trials following a long-term instruction in the block after the nutrition-video between 420 ms and 540 ms as well as between 640 ms and 1000 ms (see Figure 12C). No significant differences between the taste properties of both experiments were found for long-term instructions and the migraine video (see Figure 12D). Time-series with consecutive significant beta weights are shown in Table 4.

## 6. General Discussion

In this study, we investigated the temporal dynamics and potential influencing factors on healthy food choices using a binary food choice task in two experiments. We used two different manipulations to influence the decision-making process. In the explicit manipulation, participants were instructed before each trial to focus on either the short-term or the long-term consequences of their choices. The implicit manipulation focused on creating different mindsets. Therefore, participants watched a video clip on either the components of food and the importance of a balanced diet (food-related content) or on myths and causes of headaches and migraines (food-unrelated content) before an experiment block. Prior research has shown that both types of implicit and explicit information have an impact on dietary self-control [10,16,18,19,20,21]. Previous studies left open the question of the extent to which explicit versus implicit manipulations may differ or interact in their effects on dietary decisions. Therefore, we directly compared both potential influences in a within-subject design, which also allows for examining potential interactions. Specific instructions, as an explicit manipulation, had a significant effect on choice behavior, choice times, and the underlying decision process, which was recorded via mouse tracking. Participants’ mindset, as an implicit manipulation, showed mixed results and did not influence choice behavior or the underlying conflict as strongly as the explicit manipulation.

The instruction to consider distinct consequences in the explicit manipulation had a strong effect on dietary choices. When participants were instructed to focus on the short-term consequences, they chose the tasty option significantly more often. When they were instructed to focus on long-term consequences, they chose the healthy alternative significantly more often. This effect was found in both experiments. Clearly, the importance of health aspects is enhanced when focusing on future consequences, which facilitates the choice of a healthy option. The current mindset seems to have a minor effect on choice behavior. However, it is noteworthy that the blocked presentation of the cue led to a significant interaction between explicit and implicit manipulations. If participants deliberately focused on long-term consequences, a mindset related to the theme of a balanced diet enhanced the positive effects of the instruction to focus on long-term consequences, as indicated by a higher number of healthy choices compared to a control (food-unrelated) mindset. When participants focused on short-term consequences, no differences in choice behavior between both mindsets were found. This interaction effect did not reach significance when the cues were presented in random order, but descriptively, a similar pattern was found. These results suggest that the nutrition video enhances the positive effects of representing long-term consequences on healthy food choices, leading to a higher number of healthy food choices in the nutrition video block compared to the migraine video block.

Moreover, response times also showed a strong effect of instructions. Choices were faster if they were congruent to instructions (i.e., unhealthy choices when the focus was on short-term consequences and healthy choices with an emphasis on long-term consequences) and slower if they were incongruent to instructions (healthy choices for short-term consequences and unhealthy choices for long-term consequences, respectively). This demonstrates that focusing on the consequences of one’s choices does not only affect choice behavior but also the decision conflict. As previously described, the choice of a healthier food item and the associated rejection of a more palatable option are considered dietary self-control conflicts. However, focusing on long-term consequences compared to short-term consequences results in reduced response times for healthy choices and hence indicates a reduced decision conflict. Again, focusing on long-term consequences strengthens the importance of health aspects [34]. This reduces the subjective value difference between health and taste, resulting in less conflict when choosing the healthy option.

While this pattern of response times was found for both mindset-conditions with blocked cue presentation (i.e., Experiment 1), it was only found for choices during a food-related mindset with random cue presentation (i.e., Experiment 2). No effect on choice times was found during a mindset with a control condition. The reason why there is no difference between both mindset manipulations in Experiment 1 might be the blocked presentation of the explicit instruction cue. By using blocked instruction cues (as compared to random instruction cues in Experiment 2), we might have created a second mindset on its own since participants knew the instruction would be the same for the entire block, with stronger effects than the actual mindset manipulation. This led to a reduced self-control conflict (i.e., reduced choice times) while choosing the healthy item during long-term instructions, as well as a food-unrelated mindset. Previous research has shown that focusing on the future can also act as a general mindset and influence food choices. For example, ref. [35] used episodic future thinking (EFT) to influence food choices. They asked participants to vividly imagine future or past events related to food or in a control context and write a short text about them. During the writing task, snacks and water were offered for free consumption, and calorie intake was then calculated for each participant. They found that food-related EFT, compared to general EFT, leads to reduced food intake. In comparison to our study, the focus on the future can be seen here more as a mindset that was created before the task was processed. The constantly repeated focus on future consequences in the present study may have generated a general long-term mindset that additionally influences decisions even during our control mindset, whereas with randomized cues, as in Experiment 2, the actual mindset manipulation seems to make the difference. If participants have to reassess and consider the consequences of each decision (as in Experiment 2), a healthy mindset intervention helps reduce the conflict in choosing the healthy item, which can be seen in the reduced response times.

Further insight into the influence of the manipulations on the underlying decision conflict were obtained from the mouse tracking data. Both experiments clearly showed the predominant influence of taste properties on the decision process in trials with short-term instructions, with the significant difference between health and taste features starting earlier in the first experiment (see Figure 6 and Figure 10). Once again, this might be due to the blocked cue presentation in Experiment 1. Because participants spent an entire block focusing on short-term consequences in the first experiment, the importance of taste attributes may have been more salient (and thus considered more quickly during the decision-making process) than in the second experiment with randomized cues. This result is consistent with findings on response times, where the presentation of blocked cues led to faster decisions than the presentation of random cues [36,37]. By using the same instruction for an entire block, we might have created a mindset on its own with stronger effects than the mindset manipulation via the videos. This finding is also in line with studies from the task switching domain, where task switches typically lead to slower response times compared to task repetitions (for a review, see [38]).

Regarding trials with long-term instructions, there was no difference between the influence of taste and health properties in both experiments compared to trials with short-term instructions, where the influence of taste aspects exceeded the influence of health aspects on the angle of mouse trajectories (see Figure 6 and Figure 10). This also shows that the conflict between subjective taste and health values can be modulated by focusing on future consequences. Interestingly, although the influence of health properties does not exceed the influence of taste properties, the choice of healthy foods is still enhanced. With regard to possible interventions, this means that it is sufficient to emphasize the importance of health aspects in food selection. It appears not to be necessary to change the absolute weight of the perceived taste characteristics, but it suffices to increase the relative weight of health compared to food properties in the value integration process. Concerning the mouse data, we did not find significant differences between the two mindset manipulations, which also suggests that the mindset manipulation have a subordinate effect on the decision conflict compared to concentrating on future consequences.

Additionally, the way in which the cues were presented had a significant influence on the impact of the individual health and taste attributes on the decision process (see Figure 11 and Figure 12). Comparing the influence of the respective health and taste properties on the decision-making process between experiments (i.e., blocked versus random cues), we found that blocked cue presentation especially enhanced the weight of health attributes during the decision process in trials with long-term instructions (see Figure 11C,D), and this effect was particularly pronounced in the nutrition video condition. Again, focusing on future consequences seems to facilitate healthy food choices by increasing consideration of health aspects. Moreover, the positive effects of a long-term focus appear to be supported by repeated use, as in the blocked cue design, and when combined with a food-related mindset, as induced by the nutrition video. As expected, focusing on short-term consequences did not influence the consideration of health aspects during decision-making (see Figure 11A,B). However, one might have expected that the focus on short-term consequences would affect the processing of taste features. To the contrary, the different types of cue presentation did not influence the impact of taste features on the decision process (see Figure 12A,B). This suggests that taste characteristics are taken into account by default [15,16]. Therefore, blocked cues do not induce any additional enhancement of the taste properties for trials with a focus on short-term consequences. Unexpectedly, the influence of taste properties is enhanced by blocking compared to random cues for trials with a focus on long-term consequences following a video with food-related content (see Figure 12C). However, this does not seem to diminish the increased consideration of health issues for trials with long-term instructions and still results in an enhanced choice of the healthy option, as one can see in the resulting food choices. Speculatively, this might suggest that, despite the increased consideration of health aspects under the instruction of long-term consequences, taste is nevertheless taken into account due to its faster and more automated processing [15,39]. Moreover, a healthy decision should not come at the expense of taste in the sense of satisfaction optimization. Future research should investigate whether and how both attributes are optimized depending on the options and the exact time horizon.

In line with prior research, we showed that food choices are more influenced by taste than health, but that it is possible to influence the decision-making process towards healthier choices. For example, ref. [16] examined attentional aspects by explicitly instructing participants to consider the health versus taste aspects during dietary decisions. They found that concentrating on health aspects enhances choices of healthy food items as compared to concentrating on taste aspects. Likewise, focusing attention on the health aspects of food items has also been shown to reduce cravings for tasty but unhealthy food [17]. Ref. [10] showed that monetary incentives enhance motivation for dietary self-control behavior. When participants receive a reward for losing weight, they choose the healthy but tasty option more often and reject the unhealthy but tasty option in a food choice task compared to a condition where they respond naturally. Ref. [18] tried to modify the temporal bias favoring taste information by presenting external health-related cues in a normal-weight, overweight, and obese group. They found that presenting caloric information changed the speed of processing health information in the overweight group and promoted healthier food choices. Our results extend the findings of previous research by showing that focusing on long-term consequences facilitates healthy food choices. However, the effects of the current mindset were less pronounced in the present study. Prior research showed that adopting a healthy mindset influenced dietary decisions by choosing the healthy option more frequently [19] and choosing a smaller portion size in both adults with normal weight and obese adults [20,21]. Compared to previous studies, we used a less distinctive mindset manipulation. For example, refs. [20,21] specifically instructed their participants to focus their mindset on the health aspects of food or expected pleasure, which appears similar to our cue-based manipulation to focus attention on the long-term consequences of food choices. By contrast, our mindset manipulation was implicit in the sense that we did not explicitly instruct participants to put themselves deliberately in a particular mindset but rather attempted to induce a mindset indirectly via the content of the presented video clips. Moreover, our mindset manipulation highlighted the importance of a balanced diet, which can include sweets or convenience foods as long as they do not constitute the main part of food intake. Thus, our manipulation was more general and not specifically targeted at effects directly related to food intake, which could be the reason why our results differ from former research and our mindset manipulation did not have a greater influence on food choices. Still, our manipulations related to health aspects (focusing on long-term consequences and the short video about a balanced diet) should enhance the importance of a healthy lifestyle and therefore the health aspects of food. Hence, our results suggest that the effect of an implicit mindset manipulation may not influence the decision process as strongly as an explicit instruction to establish a particular mindset or a constant reminder at the time of choice.

### Limitations

One limitation of the present study is that the performance of the decision task requires a certain minimum number of predefined item combinations, which must be filled by the rating. However, this was necessary to create an individual stimulus set and to generate meaningful conflicts for all participants. Moreover, since the effects of mindset manipulation seem to be rather fragile, our sample size may have been too small to reliably detect these effects. Though the mindset application asks for future, more highly powered replications to be sure, the main objective of the present study was to compare the effects of explicit and implicit information on food choice. It was nevertheless shown that explicit information appears to have a greater influence on the decision and the associated conflict. Also, we have conducted our study within a design where there is a possibility that the order of the videos may have an influence. This was carried out for economic reasons and in line with previous studies [20,21]. However, our results show that we can rule out the influence of interest. Furthermore, choosing the migraine video as a control mindset may not have been the best choice, as migraines can be accompanied by nausea. While this may not have an impact on people who do not suffer from migraines, this video clip could have an impact on the food choices of people who do suffer from migraines, and hence, it is not a neutral mindset for them. To exclude this influence, the self-report questionnaire included in our study also asked about medications taken regularly. Since none of the participants reported using migraine medications or ibuprofen, we assume that none of the participants suffer from migraines on a regular basis.

## 7. Conclusions

The present study examined the influence of explicit manipulations of attentional focus and implicit priming of mindsets on dietary food choices. In line with previous research, our results show that taste attributes appear to be considered more strongly than health attributes in the decision-making process by default. However, we also showed that it is possible to enhance healthy food choices and that an explicit focus on health consequences appears to be more effective than an implicit priming of a health-related mindset. This suggests that focusing on future consequences (as explicit information) has great potential for improving dietary decision-making. By focusing on long-term consequences, the proportion of healthy food choices can be increased, and the underlying decision conflict can be reduced. Therefore, the goal of interventions should be to highlight the health aspects and reinforce their importance, which appear to work especially effectively by focusing attention on the long-term consequences of dietary choices at the moment of decision. It may therefore be beneficial to provide respective attention-directing cues at a fairly frequent and reoccurring rate, e.g., before every meal.

## Figures and Tables

**Table 1:** Consecutive time segments of significant differences between beta weights of the predictors taste and health in time-continuous multiple regression of mouse angle trajectories of Experiment 1 for all four manipulation combinations.

	Short-Term Instructions		Long-Term Instructions	
	Video: Nutrition	Video: Migraine	Video: Nutrition	Video: Migraine
Consecutive significant time period (*p* < 0.05)	[320, 1200]	[340, 1200]	-	-

Note: The numbers in brackets correspond to the start and endpoint (in ms) of consecutive time-series with a significant difference between the influence of taste properties and health properties on the mouse angle trajectories after stimulus onset. For instance, mouse angel trajectories were significantly more influenced by taste compared to health between 320 ms and 1200 ms after stimulus onset for trials with short-term instructions after watching a video about nutrition.

**Table 2:** Consecutive time segments of significant differences between beta weights of the predictors taste and health in time-continuous multiple regression of mouse angle trajectories of Experiment 2 for all four manipulation combinations.

	Short-Term Instructions		Long-Term Instructions	
	Video: Nutrition	Video: Migraine	Video: Nutrition	Video: Migraine
Consecutive significant time period (*p* < 0.05)	[610, 620] and [720, 1500]	[710, 1500]	-	-

Note: The numbers in brackets correspond to the start and endpoint (in ms) of consecutive time-series with a significant difference between the influence of taste properties and health properties on the mouse angle trajectories after stimulus onset. For instance, mouse angel trajectories were significantly more influenced by taste compared to health between 710 ms and 1500 ms after stimulus onset for trials with short-term instructions after watching a video about migraine.

**Table 3:** Consecutive time segments of significant differences between beta weights of the predictor health between both experiments in time-continuous multiple regression of mouse angle trajectories for all four manipulation combinations.

	Short-Term Instructions		Long-Term Instructions	
	Video: Nutrition	Video: Migraine	Video: Nutrition	Video: Migraine
Consecutive significant time period (*p* < 0.05)	-	[130, 280]	[430, 1180]	[410, 710]

Note: The numbers in brackets correspond to the start and endpoint (in ms) of consecutive time-series with a significant difference between the influence of health properties in Experiment 1 and health properties in Experiment 2 on the mouse angle trajectories.

**Table 4:** Consecutive time segments of significant differences between beta weights of the predictor taste between both experiments in time-continuous multiple regression of mouse angle trajectories for all four manipulation combinations.

	Short-Term Instructions		Long-Term Instructions	
	Video: Nutrition	Video: Migraine	Video: Nutrition	Video: Migraine
Consecutive significant time period (*p* < 0.05)	[390] and [470, 570]	[160, 200]	[420, 540] and [640, 1000]	-

Note: The numbers in brackets correspond to the start and endpoint (in ms) of consecutive time-series with a significant difference between the influence of taste properties in Experiment 1 and taste properties in Experiment 2 on the mouse angle trajectories.

## Data Availability

The datasets and analysis scripts supporting the conclusions of this article are openly available in the OSF repository, https://osf.io/3n42s/ (accessed on 26 December 2023).
